# A Rare Case of Chylothorax Following a Redo Laparoscopic Nissen Fundoplication

**DOI:** 10.7759/cureus.41419

**Published:** 2023-07-05

**Authors:** Waleed Sultan, Sarvesh Naik, Karthik Kondapally, Lynn Johnston

**Affiliations:** 1 Family Medicine, Conemaugh Memorial Medical Center, Johnstown, USA

**Keywords:** gastroesophageal reflux disease, gerd, chyle in the pleural cavity, laparoscopic surgery complications, large pleural effusion, hiatal hernia repair, laparoscopic nissen’s fundoplication, chylothorax

## Abstract

Gastroesophageal reflux disease (GERD) is a highly prevalent disease. Mechanical etiology, including hiatal hernia, can be resistant to empiric proton pump inhibitor (PPI) trials; Nissen fundoplication is commonly used to treat mechanical GERD. Chylothorax is a rare complication of abdominal surgeries, including anti-reflux procedures. In this case report, a 75-year-old female presented with shortness of breath following a redo laparoscopic Nissen fundoplication. Chest CT pulmonary angiography (CTPA) showed bilateral large pleural effusions that were managed by fluid restriction, repeated thoracocentesis, and chest tube insertion; the pleural fluid analysis was significant for fluid triglycerides high at 232 mg/dL which was diagnostic for chylothorax. The patient was treated conservatively. Appropriate management of chylothorax is crucial to avoid subsequent respiratory failure, immunodeficiency, and malnutrition. Chylomicrons and triglycerides in the pleural fluid can be diagnostic for chylothorax. Treatment of chylothorax includes three main approaches: controlling the cause, conservative treatment, and surgical interventions.

## Introduction

The prevalence of gastroesophageal reflux disease (GERD) is increasing with more than 30% of adults in the United States endorsing weekly symptoms [1]. Furthermore, 40% of adults with GERD report refractory symptoms that do not adequately respond to proton pump inhibitor (PPI) therapy [2]. First described in 1991 [3], laparoscopic Nissen fundoplication (LNF) has been the gold standard surgical option [4]. The main cause of iatrogenic chylothorax is thoracic surgery, including esophagectomy; It is extremely rare after abdominal surgeries like LNF [5]. It occurs due to the accumulation of milky chyle within the pleural space resulting from disruption or obstruction of the thoracic duct or its tributaries. The diagnosis depends on fluid analysis by assaying the triglyceride content and chylomicrons [6]. Early diagnosis of chylothorax is crucial to avoid further complications and persistent pleural effusion following thoracic or abdominal surgery should warrant suspicion of chylothorax [5]. Prompt management is critical to avoid possible morbidities and mortality. Here, we describe a rare case of chylothorax following an LNF procedure.

## Case presentation

A 75-year-old female with hypertension, hypothyroidism, hyperlipidemia, diabetes mellitus type 2, and a history of floppy Nissen fundoplication with mesh placement 13 years earlier presented to the ED with shortness of breath. This occurred five days after undergoing a redo laparoscopic hiatal hernia repair and Nissen fundoplication with bilateral pigtail placement for recurrent symptoms of regurgitation, progressive shortness of breath, and chronic cough. She had a moderate diaphragmatic hernia on computed tomography (CT) of the abdomen and pelvis.

**Figure 1:**
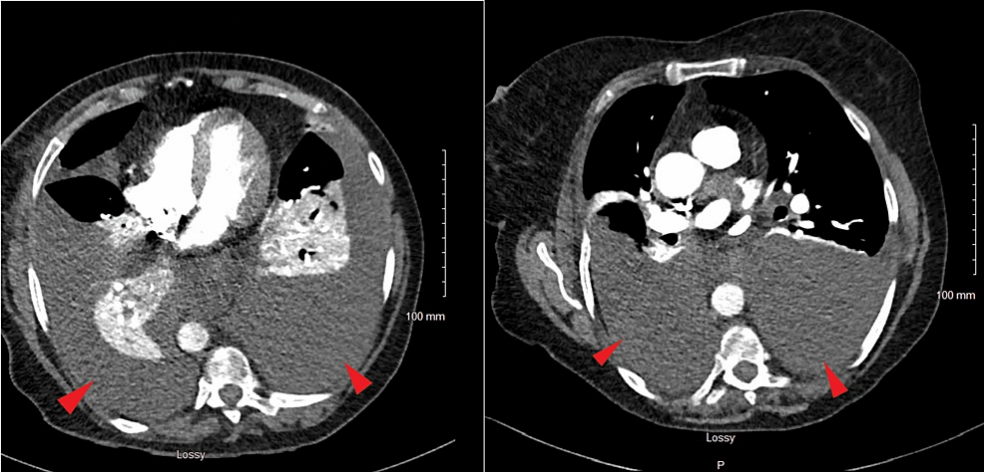
CTPA showing bilateral large pleural effusions with extensive bilateral lower lobe atelectasis; the aerated lungs are clear. CTPA: computed tomography pulmonary angiogram

After the procedure, her dyspnea progressively worsened and was associated with chest pain; however, she did not endorse any other symptoms. The physical exam revealed diminished lung sounds bilaterally. Her bloodwork was unremarkable except for a mildly elevated white blood cell count; B-type natriuretic peptide and troponin I levels were within normal range. The blood culture grew *Micrococcus luteus* in one bottle, likely due to contamination. CT pulmonary angiography (CTPA) showed large bilateral pleural effusions with bilateral lower lobe atelectasis (Figure 1).

The echocardiogram was insignificant, which ruled out an underlying cardiac cause of the pulmonary effusion. Aggressive diuresis was started, bilateral thoracentesis was performed, and 1,100 mL of cloudy fluid was evacuated bilaterally. The pleural fluid analysis bilaterally showed slightly low pH with increased protein, lactate dehydrogenase, and glucose content but had a low amylase, adenosine deaminase, and cholesterol content; it also revealed increased red cell and white cell count with abundant lymphocytes and was negative for malignant cells. The pleural fluid culture didn't grow any organisms; the right side pleural fluid analysis is shown in Table 1. The patient's symptoms were relieved, and she was discharged.

**Table 1:** Pleural fluid analysis LDH: lactate dehydrogenase; WBCs: white blood cells; RBCs: red blood cells.

Marker	First analysis	Second analysis	Reference Range
Appearance	Cloudy	Cloudy	Straw-colored, clear
Protein	2.9 g/dL	2.3 g/dL	< 1-2 g/dL
LDH	156 U/L	150 U/L	No reference established
Glucose	155 mg/dL	153 mg/dL	No reference established
pH	7.48	7.45	7.5-7.6
Amylase	<20	Not Tested	30-110 U/L
Adenosine deaminase	8 U/L	4 U/L	0-30 U/L
Cholesterol	49 mg/dL	49 mg/dL	<200 mg/dL
WBCs	2,508/cumm	2,635/cumm	1400-3700/cumm
Lymphocytes	85%	66%	18-36 %
RBCs	>20,000 /cumm	>63,000 /cumm	Nil
Malignant cells	Nil	Nil	Nil
Triglycerides	Not Tested	232 mg/dL	<110 mg/dL

**Figure 2:**
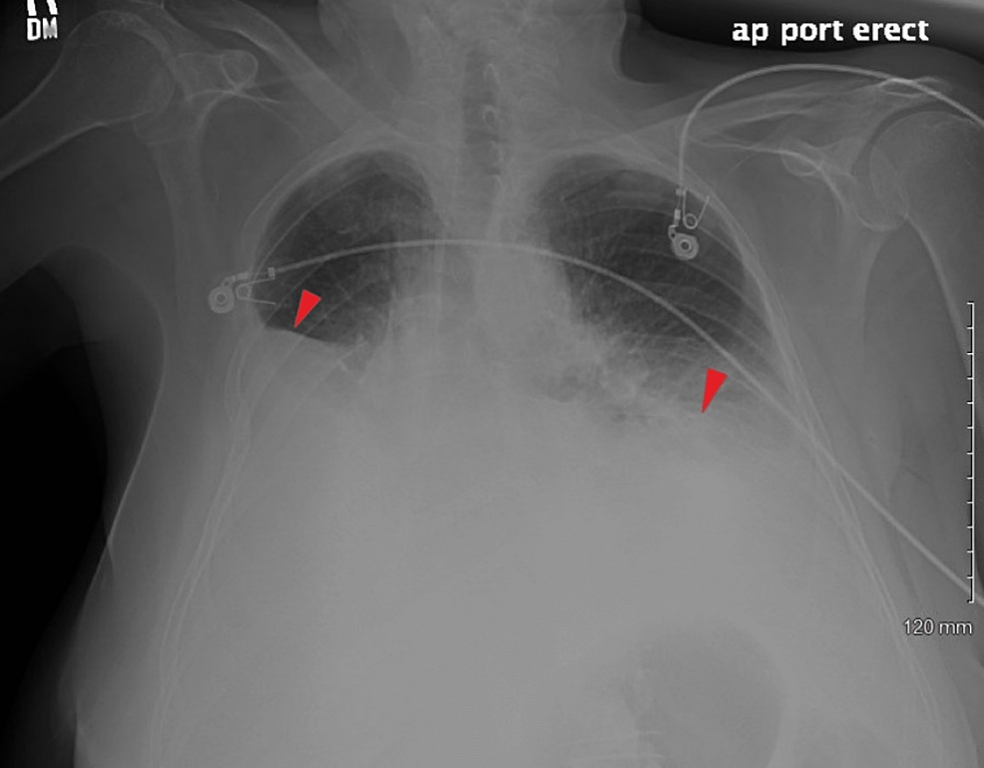
Chest X-ray showing large bilateral pleural effusions, more on the right side than the left, three days post thoracocentesis, and nine days post LNF. LNF: laparoscopic Nissen fundoplication

**Figure 3:**
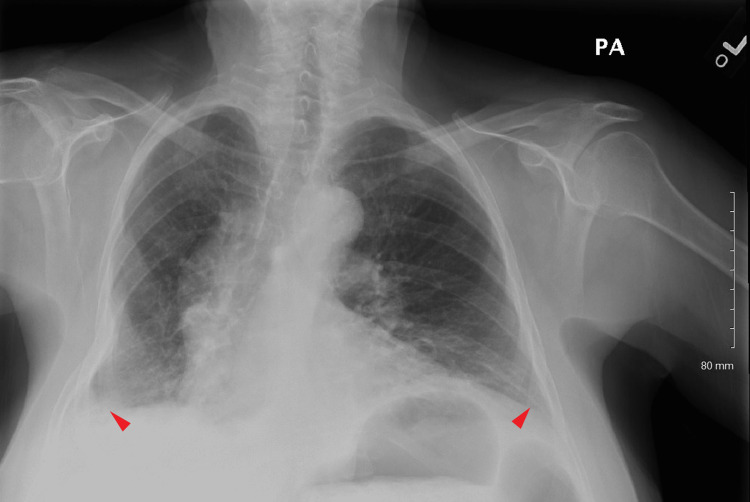
Chest X-ray showing small bilateral pleural effusions two days after discharge, 19 days post LNF LNF: laparoscopic Nissen fundoplication

However, the patient again returned to the ED two days later with shortness of breath. She had mild leukocytosis and slightly elevated creatinine of 1.3 mg/dL (baseline 1-1.1 mg/dL). Chest X-ray showed large bilateral pleural effusions, more on the right than on the left (Figure 2). The patient was hemodynamically stable but required 5 L oxygen by nasal cannula. Diuresis was initiated, thoracentesis of the right side was performed, and another 1300 mL of cloudy pleural fluid was obtained. The pleural fluid analysis was not significantly different from the initial one (Table 1). As chylothorax was suspected, the pleural fluid triglycerides were tested and were highly elevated at 232 mg/dL. A right-side pigtail chest tube was inserted into the pleural cavity and initially drained 100 mL. Conservative treatment was continued with fluid restriction, diuresis, and pleural fluid drainage. The pigtail chest tube was removed four days later. With the patient remaining asymptomatic on room air, she was discharged home on a diuretic. At follow-up, 48 hours later, the chest X-ray showed small bilateral pleural effusions that didn't progress (Figure 3); they were unchanged on repeat X-ray two weeks later, and the patient remained asymptomatic. 

## Discussion

Esophageal symptom characterization of heartburn and regurgitation has 70% sensitivity and specificity for GERD, suggesting first-line therapy of PPIs with high rates of symptomatic relief without objective testing [7,8]; however, empiric PPIs may be ineffective in the presence of other mechanisms, including obesity and hiatal hernia [9]. LNF was first described in 1991 [3] and has been the gold standard for the surgical management of GERD [4]. Typical postoperative complications of LNF include wrap migration, trans-diaphragmatic herniation, wrap ischemia with perforation, dysphagia, pneumothorax, surgical emphysema, pneumomediastinum, wound complications, recurrent regurgitation, nausea, and vomiting [10].

Chylothorax is characterized by the accumulation of chyle within the pleural space due to disruption or obstruction of the thoracic duct or its tributaries, the diagnosis of which is supported by pleural fluid triglyceride concentration greater than 110 mg/dL. Since the content of the chylous fluid is usually high in triglycerides, lymphocytes, and immunoglobulins, prolonged chylothorax causes dyspnea, immunodeficiency, and malnutrition [6]. Although chylothorax is rare after abdominal surgery, it can happen following bariatric and anti-reflux procedures [5]. 

Chyle is usually milky and can be mistaken for pus. In traumatic cases, it can be blood-stained. Chylomicrons are molecular complexes of proteins and lipids synthesized in the jejunum, and their presence in the pleural fluid is the gold standard diagnostic for chylothorax [11]. Chylomicrons can be identified on cytological analysis of fluid stained with Sudan III [6]; measuring fluid cholesterol and triglyceride levels is an alternative test. Pleural fluid with triglyceride of 1.24 mmol/L (110 mg/dL) and cholesterol < 5.18 mmol/L (200 mg/dL) is diagnostic of chylothorax [6,12].

Treatment of chylothorax can be achieved through three main approaches [11]: (i) controlling the underlying causes, e.g., sarcoidosis, (ii) conservative measures including nutrient replacement, nil by mouth, the administration of low-fat medium-chain triglycerides, which have been successful in 50% of traumatic cases (somatostatin and octreotide are useful through reducing the intestinal chyle production), and (iii) surgical treatment such as thoracotomy to ligate the damaged lymphatic ducts, with a potential role for interventional radiology in patients with persistent chyle leakage as cannulation and embolization have been successful in such cases. Lymphangiography had diagnostic and therapeutic benefits in identifying and treating leakage sites [6]. In our case, conservative treatment successfully controlled the patient's condition.

## Conclusions

Chylothorax is a rare complication of abdominal surgeries that should be ruled out in patients with postoperative persistent pleural effusion. Measuring the pleural fluid chylomicrons and/or triglyceride levels is essential to confirm the diagnosis of chylothorax. Management can be achieved through different approaches, including controlling the cause, conservative treatment with fluid restriction, diuresis, pleura fluid drainage, and surgical intervention.
